# Ten simple rules for running a summer research program

**DOI:** 10.1371/journal.pcbi.1010588

**Published:** 2022-11-03

**Authors:** Joseph C. Ayoob, Juan S. Ramírez-Lugo

**Affiliations:** 1 Department of Computational and Systems Biology, University of Pittsburgh School of Medicine, Pittsburgh, Pennsylvania, United States of America; 2 Department of Biology, Universidad de Puerto Rico, Rio Piedras, San Juan, Puerto Rico, United States of America

## Abstract

To continue to advance the field of computational biology and fill the constantly growing need for new trainees who are well positioned for success, immersive summer research experiences have proven to be effective in preparing students to navigate the challenges that lay ahead in becoming future computational biologists. Here, we describe 10 simple rules for planning, offering, running, and improving a summer research program in computational biology that supports students in honing technical competencies for success in research and developing skills to become successful scientific professionals.

## Introduction

**Fig 1:**
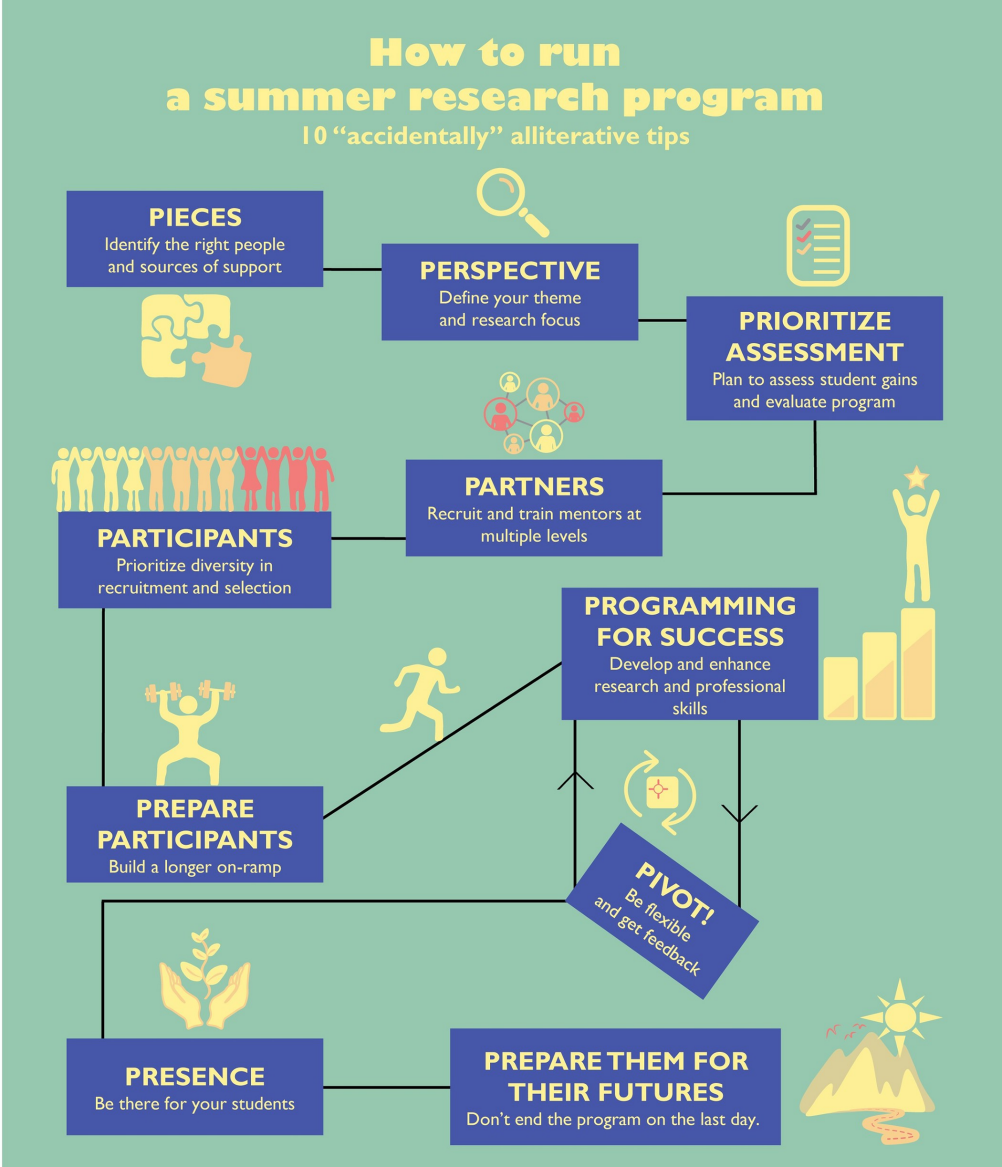
Ten simple rules for running a summer research program in computational biology. A graphical representation of our “accidentally” alliterative rules to planning, offering, running, and improving summer research experiences in computational biology.

Science is a rapidly evolving field, and there are few areas that are growing more rapidly than computational biology. As such, our educational efforts, including those outside of the traditional classroom, must keep pace to continue to advance the field and fill the constantly growing need for new computational trainees who are well positioned for success in both academia and industry. While there is a rise in traditional undergraduate programming and majors, many students still do not have access to hands-on research training in computational biology at their home institutions. Summer research programs, like the National Science Foundation’s Research Experiences for Undergraduates (REU) program and others, have been providing these opportunities to cohorts of students by supporting their travel to and participation in full-time, multi-week programs working alongside faculty for an immersive research and training experience. These programs have helped students learn about and explore new scientific disciplines, discern if graduate school is the right path for them, and prepare for future successes in academic and industry research and other careers in science, technology, engineering and mathematics (STEM). Importantly, many of these programs have also focused on giving opportunities to students who hail from groups that are underrepresented in the sciences. These early interventions are one important step in positioning students to be prepared to pursue research in interdisciplinary fields, which often needs advanced planning to ensure that students have the necessary training in both the life and physical sciences as well as computational and quantitative areas. To pave the way for the next generation of discoveries and continue to cultivate the field of computational biology [1], we must make training the next generation of computational biologists a priority. Experiential programs like REUs play an important role in preparing students interested in computational research, and we encourage more departments and graduate programs to offer summer research experiences to undergraduates who will become the next generation of computational scientists. Here, we outline 10 “accidentally” alliterative rules for running a summer research program in computational biology (Fig 1). This article will be of interest for department chairs, program directors, undergraduate research coordinators, and anyone else interested in providing research experiences in computational biology or related fields to promising undergraduates.

### Rule 1: Pieces—Identify a leadership team and build toward obtaining institutional and/or grant support

Formalized summer undergraduate research programs don’t have to be built overnight. They can be grown organically from existing mentor–mentee relationships, research collaborations, and/or specific research interest groups, such as multi-lab journal clubs and cross-department working groups. For sustained growth, however, there are a few important catalysts that can accelerate the process. Primary among them is having a dedicated faculty member or team, who is willing to spearhead the effort to grow and sustain the program. If a department or unit is to commit to a program, then providing additional administrative help specifically for the program will facilitate its planning, coordination, and operation. Since this can be a considerable effort, protected time and formal recognition of the effort at the department and university levels will further enable the program director, whether they are on the tenure-track or not, to devote the necessary time for building the framework of the program, recruiting faculty mentors at their own institutions, finding partners at neighboring institutions and beyond, connecting to existing support networks, e.g., the BIO REU that supports REU programs in biology [2], and procuring additional money to sustain the program and fund the students and their work. While grant money for these programs can be competitive, piloting a program, even with only a few students who are supported by institutional funds, and being able to demonstrate its effectiveness through the success of its students, can help convince review panels that a strong infrastructure is in place to support a larger program that will be of great benefit to nascent scientists.

### Rule 2: Perspective—Define your theme or research focus

A defined research theme provides a strong program identity, which will guide its development and facilitate student recruitment. Having a clear research focus also aligns well with funding agency guidelines for programs; if you are considering applying for grant support, selecting and building upon a unifying theme early in the process will be an advantage for proposal preparation. A theme can be broad, such as “multiscale modeling of biological systems,” which might incorporate several research areas across various biological scales, or specific, such as, “comparative genomics of gut microbiomes.” Of course, if one has a proclivity for generating memorable or groan-inducing acronyms for scientific programs, this is a ripe opportunity to do so [3]. Other advantages of a theme are that it facilitates the establishment of a common language for participants as well as providing immediate connections and commonalities to help them connect with each other and the associated faculty.

### Rule 3: Prioritize assessment and evaluation—Make a plan to assess student gains and evaluate the implementation of the program

While many computational biologists would rather do double committee duty than have to think about assessment and evaluation, we would urge program directors to incorporate these relatively painless and essential elements into their programs from the beginning. If you are completely averse to this, one could always scrape up some cash and pay for an external evaluator to help with the implementation and analysis. If you are a braver soul, then fear not; there are some existing tools and strategies that can help guide you.

One overarching framework that can help you put together a comprehensive plan for your program is the backward design approach that is used for curriculum development [4,5]. The first step would be to reflect on what the learning objectives that you have for your students are and what gains you expect them to make by the end of the program (summative) and at various checkpoints along the way (formative). Similarly, think about what you’d like for the program to achieve with respect to outcomes, quality of enrichment activities, student satisfaction and attitudes, among other metrics. Next in the backward design process would be to decide on ways that you will assess the various goals and objectives that have been set. For this, one does not have to reinvent the wheel as there are numerous validated instruments that can be easily implemented, such as the Survey of Undergraduate Research Experiences (SURE), the Undergraduate Research Student Self-Assessment (URSSA), and the Entering Research Learning Assessment (ERLA) and other instruments through the Center for the Improvement of Mentored Experiences in Research (CIMER) Assessment Platform [6–10]. Finally, after the goals, objectives, and assessment have been set, create a schedule of program activities that aligns with these elements.

If you do catch the assessment bug and want to start assessing everything in sight, we would advise caution to limit the number of surveys given to students to reduce survey fatigue that may affect the quality of student input. Also, don’t overlook the power in the simplest of surveys, such as weekly check-ins with students and brief questionnaires that quickly take the pulse of the students and the program and allow for any necessary course corrections in the middle of the program. If longer-term student outcomes will be tracked, e.g., collecting data on how many students pursue graduate degrees in STEM, having an established method for connecting with students after the program ends will greatly aid this endeavor.

### Rule 4: Partners—Recruit and train mentors at multiple levels and from multiple disciplines

While the students should be at the heart of the program, any successful implementation needs a strong support system around it. Faculty will likely serve as the primary mentors for students, and a deep mentor pool can be built by identifying faculty outside of your unit who are working in areas related to the program theme. For example, there are many computationally focused scientists in biology, chemistry, physics, and engineering departments that can be recruited to build an interdisciplinary group of potential mentors. The contributions of postdoctoral fellows, graduate students, technicians, and other personnel should also not be overlooked. Incorporating more senior trainees in the training and mentorship of undergraduates not only reduces the work burden of already stretched PIs, but also yields many other positive student outcomes, which are even greater in closed mentoring triads that involve undergraduates, senior trainees, and faculty all interacting directly [11]. Working with more junior trainees also helps these more senior trainees get crucial early mentoring and teaching experiences that are not typically taught in academia. Directly incorporating a larger number of mentors not only expands the community in which the undergraduates are trained, but also helps to build a positive culture of mentoring in the host department or unit.

Recruited faculty and near-peer mentors also need support and guidance, especially if they haven’t worked with undergraduate researchers before. Students enter research experiences with different levels of discipline-specific familiarity, sets of skills, backgrounds, and perspectives that make a one-size-fits-all approach ineffective. Making mentors aware of the diverse and unique experience levels of students can help them tailor an appropriate training arc that is focused on growth. Formalized and tested training programs for mentors, such as those based on the curriculum in the publication *Entering Mentoring*, can also be implemented to equip both new and more senior mentors with essential mentoring skills, approaches, and competencies and provide a venue for them to practice these skills and share their experiences with each other [12]. Mentor training workshops as well as train-the-trainer programs for program directors or others interested in offering these workshops are available from CIMER [13] and the National Research Mentoring Network (NRMN) [14], both of which have played central roles in developing the training and implementation programs for mentor and mentee training workshops.

Another important aspect of mentor support is recognition for their time and effort. While it would be wonderful if protected time or financial compensation could be provided for mentoring, there are other ways of recognizing and appreciating the time that is invested in service to trainees. Including mentoring activities in faculty performance evaluations and tenure and promotion decisions can add much value to these efforts. Excusing faculty from dreaded committee work can be another way to incentivize participation.

### Rule 5: Participants—Prioritize diversity in recruitment and selection

Computational biology is an interdisciplinary endeavor in which having different perspectives to address complex problems is essential. While the field is rich in academic diversity, it is lacking the racial, ethnic, and gender diversity to embrace the full potential of the STEM community [15–21]. Therefore, recruitment efforts should be designed to cast as wide a net as possible to include students from a broad range of backgrounds and experiences. This will include leveraging the professional networks of your program community, reaching out to institutions in other geographic regions, and considering students from different institution types, especially those that do not have a focus on research. Diverse academic trajectories, including those of students who are returning to higher education later in life and students from community colleges, should be considered since they represent a wealth of talent and experiences that are often overlooked.

Key resources for recruitment include national conferences that showcase the research of undergraduate students, such as the Annual Biomedical Research Conference for Minoritized Scientists (ABRCMS), the American Indian Science and Engineering Society (AISES), and the Society for the Advancement of Chicanos and Native Americans in Science (SACNAS) [22]. These venues offer unique opportunities for recruitment of undergraduate minority scientists that are already engaged in research. Online resources, such as PathwaysToScience.org from the Institute for Broadening Participation and the American Association for the Advancement of Science (AAAS) Entry Point! program, are other ways to connect to underrepresented students and students with disabilities, respectively [23]. Furthermore, building and maintaining relationships with partners and partnering institutions with similar or closely aligned research interests will help ensure a continuous flow of talented applicants.

### Rule 6: Preparation for participants—Build a longer preprogram on-ramp

There can be a lot of anxiety at the beginning of a summer research experience, especially for students who are leaving their support network and coming into a completely new environment. Compounding this is the relatively short duration of most summer research experiences (8 to 10 weeks), which can add additional pressure on students who may feel a constant urgency and undue pressure to produce results. To address these concerns, having students come to the program prepared and knowing what to expect from the summer can ease some fears, help them hit the ground running, and make it a more enjoyable experience for everyone. Hosting virtual icebreakers with students and, if possible, 1 or 2 additional virtual meetings before the official start of the summer program is a great way to get the ball rolling. These meetings can serve as ways for students to get familiar with each other, their mentors, and the leadership team of the program and ask questions that may help ease the preprogram jitters. Incorporating mentors in these conversations, or in separate one-on-one meetings, can also help with getting students acclimated with whom they will be working and the project that they will undertake. These initial meetings can also be a good way to let students know what software and/or programming languages they will be using during the summer so that they can start downloading and installing on their personal computers and read up documentation and/or complete free online tutorials, such as those available on codecademy.com, before day one [24].

### Rule 7: Programming for participants—Develop and enhance research and professional skills for student success

Multiple opportunities for participants to gain and refine skills will be useful in their advancement toward becoming computational biology researchers and leaders in the field. An effective and impactful summer research program focuses on the development of skills in 2 broad categories—research skills and professional skills. For computational biology–oriented research, some of the critical research skills include fluency in one or multiple programming languages and familiarity with data analysis pipelines. An important consideration is that incoming students may have different levels of experience with computational skills at the beginning of the program. Therefore, devoting time during the first days to teaching, refreshing, and practicing scripting, programming, and the management, analysis, and visualization of data is a great way to meet students where they are and provide the necessary tools for students to succeed during the summer and beyond. One might consider using existing curricula (e.g., Software Carpentry) or developing one that is tailored to the research theme of the summer program [25]. In addition to learning about new tools, it is critical for students to gain exposure to current trends in various realms of computational biology. This can be achieved through journal clubs and research seminars from faculty members, postdocs, and graduate students who are integrating computational tools into biological research.

Equally important are opportunities within the summer program for students to socialize with other scientists, create meaningful connections to peers and mentors, and get to see what the life of a scientist is like. These activities will increase the chances of retaining students within the scientific enterprise, a key measure of success for a summer program [26]. There are multiple ways to go about this deliberately and intentionally, such as setting up chats with mentors and other professionals to address how to create a good work–life balance, deal with adversity, manage mentor–mentee relationships, and navigate career transitions. For example, covering how to apply to graduate school, write a compelling personal statement, effectively communicate your research to scientific and lay audiences, and find and obtain funds for research, among others, can all complement the development of research skills and prepare students for further successes once they have left your program. Similar to the *Entering Mentoring* training curriculum for mentors, CIMER also offers the *Entering Research* curriculum, which includes a comprehensive list of activities that help develop and bolster trainees’ research and professional skills, including equity and inclusion awareness, researcher identity, independence, and confidence, and research ethics [27,28]. Lastly, inviting speakers from industry and other areas where computational biology skills are in high demand can introduce students to these additional career options and help prepare them to pursue them.

### Rule 8: Prepare to pivot—Be flexible

Global pandemics and natural disasters often rear their heads to remind us of the long-held maxim that the best-laid plans can go awry, or as boxing champion Mike Tyson once put it, “Everyone has a plan until they get punched in the mouth.” Be prepared to pivot to a hybrid or fully virtual offering of your program if necessary. This seems like a daunting task, yet our new familiarity with remote work and the experience and lessons learned by colleagues who have had to pivot to remote make this process more manageable. In 2020, many REU programs transitioned to online offerings in response to the COVID-19 pandemic [29,30]. A large portion of these programs included computational projects, which were more amenable to an online format than lab or field-based experiences. Hence, don’t be afraid to use lessons learned by others to ensure that your program keeps running despite the circumstances or to include students who are not able to travel, due to physical limitations or family obligations, to participate in a program. Furthermore, incorporating remote work tools into the structure of a program can even save time for in-person programs as they minimize having to run around campus between meetings, allow for geographically distributed collaborations, and incorporate elements of novelty, such as Virtual Reality, which is becoming more utilized in computational biology research as a simulation, visualization, and educational tool [31–33].

### Rule 9: Presence—Be there for your students

There are multiple role models, coaches, advocates, and mentors that intervene and contribute to the scientific and professional development of students, yet it is very useful to have an individual or a small group of individuals that support students in bringing all these pieces together. This central connection point for students, which typically is the program director or leadership team, oversees all the activities of the program, helps the students cohesively weave together the many events that happen throughout the summer, and plays an instrumental role in considering the development of the whole student. This central hub helps to initiate, stimulate, and maintain communication between the student, mentors, and other connections that will enhance the student experience. This person also looks out for more opportunities for students to sustain their growth even after the program has ended (see Rule #10). Because of the unique vantage point, it is critical for these individuals to be available and engaged with the students throughout the summer, focused on having quality interactions and attentive to identifying and addressing any problems as they arise.

### Rule 10: Prepare them for their futures—Don’t end the program on the last day

Summer research programs are a springboard to launching successful scientific careers. They are not an end, but a beginning. Throughout the summer experience, preparing students for the next steps in their career transitions, particularly those that are more proximal to them, i.e., post-baccalaureate positions, fellowship applications, graduate school, and nonacademic research jobs to name a few, is key. Create opportunities for students to network with current graduate students to talk about the undergraduate-to-postgraduate transition. Connect them with program and department alumni in careers outside of academia to help expand their networks into industry, government, nonprofits, and other realms. Stay connected to your students via email and social media. Using tools such as Slack or Discord will allow you to maintain open channels of communication even after the program ends. These are good avenues to share announcements about opportunities and openings for programs and jobs with your students. Giving feedback on personal statements and fellowship applications is another way that you can remain in service to your students to provide much needed insight and help them with next stage applications. Offering waivers for graduate program application fees and whispering in the ears of the admissions committee about your great students can be effective methods to bring program alumni back to your institution.

## Conclusions

In this article, we provide a list of 10 simple rules that are focused on the development of summer research programs to train the next generation of computational biologists. If we were to distill this list down to one point, it would be to put the students and their needs first. Students are the reason why these programs exist, and this fact should be reflected in all the program’s activities and events. Summer research experiences have long been used by many STEM disciplines to train rising researchers and recruit the best talent into graduate programs. The time is ripe for computational biologists to embrace the benefits of summer research programs and create more of these opportunities that reflect the uniqueness of this boundary-spanning field. While these rules offer suggestions on running a program, ultimately, your program will take on its own identity that best serves your students and the environment in which they will learn. Hence, we fully expect that in another 5 years, others will write a sequel to this article with an additional set of 10 rules to make summer programs in computational biology better… we just ask that you use a different letter for your “accidental” alliteration.
